# A Novel Compute-and-Forward Relaying Method for Multi-Antenna Wireless Relay Networks

**DOI:** 10.3390/e25111512

**Published:** 2023-11-03

**Authors:** Xuan Yang, Jiaqi Yan, Yonggang Xu, Desheng Wang, Gang Hua

**Affiliations:** School of Information and Control Engineering, China University of Mining and Technology, Xuzhou 221116, China; xuanyang@cumt.edu.cn (X.Y.); jiaqiyan@cumt.edu.cn (J.Y.); tb18060007b0@cumt.edu.cn (D.W.); ghua@cumt.edu.cn (G.H.)

**Keywords:** compute-and-forward, wireless relay network, successive extended computation, matrix projection algorithm, rank failure

## Abstract

Compute-and-Forward (CoF) is an innovative physical layer network coding strategy, designed to enable receivers in wireless communications to effectively utilize interference. The key idea of CoF is to implement integer combinations based on the codewords from multiple transmitters, rather than decoding individual source codewords. Although CoF is widely used in wireless relay networks, there are still some problems to be solved, such as rank failure, single antenna reception, and the shortest vector problem. In this paper, we introduce a successive extended CoF (SECoF) as a pioneering solution tailored for multi-source, multi-relay, and multi-antenna wireless relay networks. First, we analyze the traditional CoF, and design a SECoF method combining the concepts of matrix projection and successive interference cancellation, which overcomes the problem of CoF rate tending to zero and rank failure and improves the network performance. Secondly, we obtain an approximate solution to the integer-value coefficient vectors by using the LLL lattice-based resolution algorithm. In addition, we deduce the corresponding concise formulas of SECoF. Simulation results show that the SECoF has strong robustness and the approaches outperform the state-of-the-art methods in terms of computation rate, rank failure probability, and outage probability.

## 1. Introduction

As a critical component of the Internet of Things, wireless sensor networks (WSNs) have emerged as a significant area of interest due to their broad applicability in various domains such as military operations, national defense, coal mine management, etc. [1]. However, the practical deployment of WSNs often faces challenges related to the suboptimal communication environment between sensors, which may result in the target not receiving effective information. To mitigate this problem and improve the reliability of information transmission from sensor nodes, the use of wireless relay techniques has been proposed. This approach allows WSNs to maintain effective operation even under harsh environmental conditions [2].

The Compute-and-Forward (CoF) strategy has emerged as an innovative and effective relay-forwarding method that has received considerable attention in the study of wireless relay networks [3,4]. Unlike traditional wireless relay networks, which view interference as an obstacle to be avoided, CoF uses a forwarding approach based on physical layer network coding to efficiently handle interference and achieve high-rate, reliable communication even in the midst of adverse communication environments by exploiting communication interference between nodes [5]. The core concept of CoF is to try to exploit reliable decoding, i.e., computation, and deliver an integer linear combination of transmitted messages to the destination, i.e., forwarding. The rate at which a relay decodes a received equation is called the computation rate, and the performance of a Compute-and-Forward scheme is given by it. The destination can solve the linear system of equations to recover the desired message by receiving enough equations and their corresponding equation integer value coefficient vectors (ICVs) rather than recovering individual messages. This method effectively solves the interference control problem and exploits the interference in the network to achieve higher communication rates between nodes. The CoF uses nested lattice codes as the basic network coding method [6], the transmitters map the message to the lattice according to the coding of nested lattice codes, and the transmission between nodes is performed by using lattice points; this method ensures that the structure of the computed algebra remains a valid codeword. Due to the characteristics of physical layer network coding and lattice code, CoF is significantly better than existing relay forwarding methods with certain interference, such as Amplify-and-Forward (AF) [7] and Decode-and-Forward (DF) [8,9].

Despite the promise of the Compute-and-Forward (CoF) framework, there are several obstacles that require further research and development. First, the problem of rank failure poses a significant challenge in CoF [10]. In the framework, each relay aims to optimize its transmission rate by selecting independent correlation coefficients. However, this may result in the destination node receiving equations with linear correlation, leading to incorrect decoding or the rank failure problem. This problem hinders the efficient message recovery from the transmitters. Second, the design of integer coefficient vectors (ICVs) effectively translates to finding the shortest vector—an NP-hard problem. This complexity calls for the development of an efficient and less complex method for ICV search, with the goal of maximizing the computation rate of the relays. Third, the traditional CoF scheme is complex due to the dense deployment of transmitters and high complexity of relays in wireless sensor networks [11]. The task of designing a new CoF scheme that can reduce this complexity while improving network performance is a significant challenge in itself.

CoF is a relay communication strategy that is suitable for a variety of application areas, especially when there is a need to improve the performance of the wireless network, reduce transmission overhead, or increase data reliability. For example, in a coal mine wireless sensor network, first of all, it is usually necessary to monitor various parameters, such as temperature, humidity, gas concentration, etc. However, due to the long distance of the coal mine tunnel, more relay nodes need to be deployed to realize communication transmission; therefore, CoF can be applied in coal mines. Secondly, the coal mine environment usually has a complex geological structure and more underground signal interference, so the use of CoF can effectively improve communication efficiency and reduce interference. Finally, mine wireless sensor networks usually operate in resource-limited environments, so efficient data transmission strategies are needed. CoF algorithms can be used in relay nodes to help reduce the communication overhead between sensor nodes and extend the network lifetime.

In this paper, we propose a new CoF method, SECoF, for wireless relay networks. Traditional CoF is prone to rank failure, leading to degradation of transmission quality. In addition, it is a difficult task to find the best ICV solution. Thus, the SECoF uses matrix projection and successive interference cancellation (SIC) technique to optimize the CoF algorithm to solve the rank failure, and also proposes the use of the Lenstra–Lenstra–$Lov\overset{´}{a}sz$ (LLL) algorithm [12] to solve the difficulty in finding the approximate solution ICVs process. The SECoF algorithm effectively reduces the rank failure probability at the destination and maximizes the transmission rate in a multi-source, multi-relay, multi-antenna wireless relay network.

The main contributions of this paper are as follows.

(1) We focus on the extension of the CoF scheme to multi-antenna nodes. Through the optimized CoF scheme, the destination node overcomes the class failure problem and obtains an approximate solution of ICVs using the LLL algorithm, which effectively reduces the complexity of the network computation and makes the algorithm more general.
(2) We propose a new CoF method, SECoF, in multi-source multi-relay multi-antenna networks. The SECoF exploits matrix projection to eliminate the correlation between equations and reduce the complexity of the algorithm. In addition, SECoF proposes an analog of the SIC technique, which overcomes the problem that the minimum rate may tend toward zero and rank failure. Therefore, relays have a higher forwarding rate and generality. Meanwhile, this paper derives the related formulas and provides the pseudo-code framework.
(3) To better reflect the rationality of the methods, this paper sets up a large number of comparative experiments by considering variables such as signal-to-noise ratio (SNR), the number of transmitters, the number of relays, and so on. The comprehensive performance is tested and compared with the currently popular relay forwarding methods. The SECoF method proposed in this paper effectively improves the computation rate of relays, reduces the outage probability, and has strong robustness.

The remainder of this paper is organized as follows. In Section 2, this paper briefly describes the background of CoF and its derived related work. In Section 3, the system model is presented. In Section 4, the paper presents the proposed SECoF method. Section 5 includes analytical simulations of the performance of the proposed methods and the results of numerical comparisons with other state-of-the-art relay network methods. In Section 6, conclusions and future work are described.

## 2. Related Work

In recent decades, several typical theories and extensions on CoF have been proposed, which can be classified based on forwarding strategy optimization and finding the relevant optimal integer vector and framework optimization. The forwarding strategy optimization mainly overcomes the problem of rank failure to make the destination node decode successfully. The search for the associated optimal integer vector mainly solves the shortest vector problem and reduces the acquisition complexity. The optimization of the CoF framework mainly increases or optimizes the overall network structure by optimizing the lattice code, thus reducing the network redundancy. It is an important research area in the design of multi-relay multi-user wireless networks. In [13], a hierarchical power amplifier algorithm is proposed, which is applied to a multi-relay multi-user wireless network, and the transmission performance of the NOMA-based downlink relay network improves the throughput. In [14], a new efficient scheme combining the use of cooperative diversity and multiuser diversity is proposed, which reduces the amount of channel estimation and helps to improve the performance of the network.

Forwarding strategy optimization is an important branch of CoF (see Table 1). In [15], a new scheduling mechanism (SCoF) is proposed. The SCoF proposes an asymptotically optimal, polynomial-time algorithm that allows the transmitting nodes to have better coding opportunities, thus maintaining the superior CoF capability without degrading with the increase in network size. In [16], a scheme based on vector quantization and computational forwarding relaying technique is proposed based on MMSE and LSE methods by analyzing the performance in Additive White Gaussian Noise (AWGN) and Rayleigh fading channels; these methods improve the efficiency in wireless relay networks. However, this method does not consider the rank failure problem. To solve the rank failure problem, some effective methods have been proposed. In [17], a discrete computational forwarding scheme DCMF is proposed, which overcomes the difficulty of having no central coordinator in a multi-relay network and effectively improves the computation rate. However, this scheme is not applicable to single-antenna relaying. To solve the single-antenna relaying problem, an efficient CoF solution is proposed in [18]. By introducing two new methods, Ext-CM and Suc-CM, this solution not only solves the rank failure problem but is also applicable to multi-antenna relaying networks. A new slow block fading Gaussian multiple access relay channel scheme is proposed based on CoF [19], which requires only the receiver to obtain CSI, has no number required for the transmitters and increases the transmission rate. In [20], for the coding structure in multi-source multi-relay networks, an overlapping chunked code (OCC) design method OCC/CF is proposed, which utilizes the chunking idea and uses random linear packet codes to achieve network overhead reduction.

The shortest vector for the design of optimal integer equations in CoF is an important research topic. In [21], a closed-loop solution using quadratic programming and the Lagrange multiplier method is proposed, and the suboptimal solution is obtained by a successive quantization algorithm. In [22], a new lattice coding is introduced to solve the shortest vector problem by optimizing the computation rate using a cooperative communication strategy. The algorithm is more efficient than the current most efficient exponential mnemonic method and has great superiority. For complex-valued channels with complex integer lattices, a low polynomial complexity algorithm for solving the optimal solution of complex scenarios and a simple linear search algorithm that balances performance and complexity are proposed in [23]. In [24], an exact polynomial complexity reduction algorithm is proposed, which can effectively reduce the complexity by reducing the number of candidate vectors. Based on this, ref. [25] effectively reduces the overall complexity and improves the efficiency of [24] by further optimizing the vectors. In [26], an iterative algorithm for finding the optimal coefficient vector in the Gaussian integer domain using an improved Schnorr–Euchner search strategy is proposed.

Optimization for the CoF framework is an important branch of research on CoF. In [27], a computationally compressed forwarding framework CCF is proposed, which takes advantage of the fact that lattice codewords have a correlation in the framework of CoF, and adds a process of compressing codewords in the computation and forwarding process to reduce the relay forwarding information rate, thus improving the network performance and reducing the network redundancy. However, the framework does not prove the impact of the number of compressions on the network to obtain the optimal network performance. In [28], a more general compression framework GCCF based on CCF is proposed. The method compression function involves the selection of message segments over a finite domain and proves that the minimum compression is the optimal performance, but the framework is only suitable with single-antenna relay networks that have some limitations.

## 3. System Model

### 3.1. Notational Conventions

Let R be the real field, and Z be the ring of integers. Lower and upper boldface letters are used for column vectors and matrices, respectively, e.g., $h\in R^{L}$ and $H\in R^{M\times L}$. For the vector h, we write $∥h∥$ denotes the $\ell^{2}$-norm of h, i.e., $∥h∥≜\sqrt{\sum_{i=1}^{n}h_{i}^{2}}$. For any matrix H, we denote the transpose by $H^{T}$.

### 3.2. Channel Model

**Figure 1:**
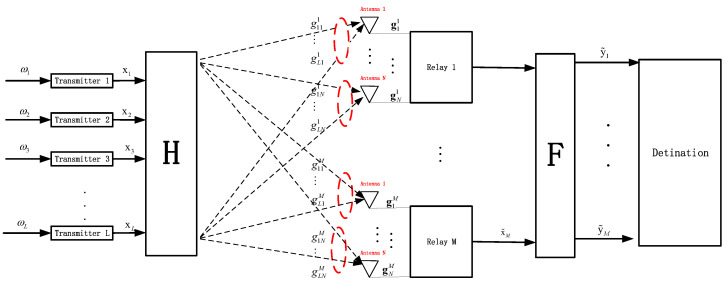
System model.

We consider the problem of maximizing the CoF computation rate and combating rank failure problem in a multi-antenna wireless relay network, which includes *L* transmitters, *M* relays, each equipped with *N* antennas, and the transmitters and destination both use a single antenna to send and receive information, respectively. The wireless relay network is depicted in Figure 1.

In this paper, we assume that all channel vectors are real-valued, there is no direct link from the transmitter to the destination, the relay has a suitable time slot to find the coefficients used to maximize the effective rate and only know its own channel state information (CSI), and the noise is independent from each other among the relays. The reasonableness of the assumption is explained below. First, the CoF algorithm receives and processes real-valued channel vectors at the relay node so the analysis problem can be simplified. Secondly, the CoF algorithm is a strategy widely used in wireless communications. This is due to the fact that wireless channels usually introduce noise and interference, whereas CoF can help to reduce transmission errors and improve network performance, hence the use of wireless channels. Finally, the selection of suitable time slots for data processing is a critical task in communication systems. CSI (channel state information) is an important piece of information for evaluating the quality of a channel. It is assumed that suitable time slots can be found for data transmission and processing as well as easy access to CSI. Therefore, the above assumptions are used in this paper.

The transmitter *ℓ* aims to transmit the information to the destination at the maximum computation rate. Each transmitter ℓ∈L has a message vector $w_{\ell }\in F_{q}^{b}$, and ℓ=1,2,…,L with length *b*, where the L represents the set of transmitters, *q* represents prime number, the prime field is $Z_{q}=\left\{0,1,\ldots ,q-1\right\}$, and it is assumed that it has equal probability on the prime size finite field. The transmitter *ℓ* utilizes a lattice encoder with a power constraint $P_{e}$ and maps the message $w_{\ell }$ from a finite field to length-ω codewords by a nested lattice encoding method $\Theta_{\ell }$: $Z_{q}^{b}\to R^{\omega }$.

**Definition** **1.** *(Channel Model with single antenna): Each relay m∈M observes a linear combination of the signal with noise through the channel can be expressed as,*

(1) $$y_{m}≜\sum_{\ell =1}^{L}h_{m\ell }x_{\ell }+z_{m}$$

*where $x_{\ell }\in R^{\omega }$ with the power constraint $\frac{1}{\omega }{∥x_{\ell }∥}^{2}\leq P_{e}$ is the transmitted codeword from the transmitter ℓ∈L. $h_{m\ell }$ is the real-valued channel gain on the link between the transmitter ℓ and relay m, the $z_{m}\in R^{\omega }$ is the noise vector with entries being Gaussian, i.e., $z_{m}∼N\left(0,I\right)\in R^{\omega }$. Let $h_{m}={\left[h_{m1},h_{m2},\ldots ,h_{mL}\right]}^{T}\in R^{L}$ be the channel vector, and $H=\left[h_{1},h_{2},\ldots ,h_{M}\right]{}^{T}\in R^{M\times L}$ is the channel matrix, i.e., the kth row of H is $h_{k}^{T}$.*

**Definition** **2.** *(Channel Model with multiple antennas): The nth antenna of relay m observed through the channel as a linear combination of signal and noise can be expressed as,*

(2) $$\begin{array}{c} {\tilde{y}}_{n}^{m}≜\sum_{\ell =1}^{L}g_{\ell n}^{m}x_{\ell }+z_{n}^{m} \end{array}$$

*where the m∈M, the n∈N, and $g_{\ell n}^{m}$ denotes the signal channel coefficient received by the nth antenna of relay m from transmitter ℓ; meanwhile, $g_{n}^{m}={\left[g_{n1},\ldots ,g_{nL}\right]}^{T}\in R^{L}$ denotes the signal channel coefficients received by nth antenna, $G^{m}=\left[g_{1}^{m},\ldots ,g_{N}^{m}\right]\in R^{L\times N}$ denotes the signal channel coefficients matrix received by the relay, and the noise $z_{n}^{m}\in R^{\omega }$.*

**Definition** **3.** *(linear combination of message): The relay m goal is to recover a linear combination of messages $u_{m}$. The $u_{m}$ is a linear combination of messages $w_{\ell }$ with an integer vector $a_{\ell }$, where ℓ=1,…,L, i.e,*

(3) $$\begin{array}{c} u_{m}=\sum_{\ell =1}^{L}a_{\ell }w_{\ell } \end{array}$$

*where $a={\left[a_{1},\ldots ,a_{L}\right]}^{T}\in Z^{L}$ is referred to as the ICVs corresponding to equation $u_{m}$.*

### 3.3. Single Antenna CoF Computing Scheme

Nazer and Gastptar provide a computing scheme using nested lattice codewords transmitted over real or complex channels, i.e., the conventional CoF. The relay *m* uses a linear combination with noise received and then chooses a scale coefficient $\alpha_{m}\in R$, the ICVs $a_{m}={\left[a_{m1},a_{m2},\ldots ,a_{mL}\right]}^{T}\in Z^{L}$, and decodes the lattice equation from $\alpha_{m}y_{m}$, so that the decoder has the equation can be written as,

(4) $$\begin{array}{cc} \alpha_{m}y_{m} & =\alpha_{m}\sum_{\ell =1}^{L}h_{m\ell }x_{\ell }+\alpha_{m}z_{m}\\ \; & =\sum_{\ell =1}^{L}a_{m\ell }x_{\ell }+\sum_{\ell =1}^{L}\left(\alpha_{m}h_{m\ell }-a_{m\ell }\right)x_{\ell }+\alpha_{m}z_{m} \end{array}$$

where the $\sum_{\ell =1}^{L}a_{m\ell }x_{\ell }$ is the codewords, and the equation $\sum_{\ell =1}^{L}\left(\alpha_{m}h_{m\ell }-a_{m\ell }\right)x_{\ell }+\alpha_{m}z_{m}$ is the effective noise.

We denote the effective noise variance $\sigma^{2}\left(h_{m},a_{m},\alpha_{m}\right)$ for CoF as follows,

(5) $$\begin{array}{cc} \sigma^{2}\left(h_{m},a_{m},\alpha_{m}\right) & ={∥\alpha_{m}∥}^{2}+P_{e}{∥\left(\alpha_{m}h_{m}-a_{m}\right)∥}^{2} \end{array}$$

The computation rate region is achievable as follows ([3], Theorem 1),

(6) $$R\left(h_{m},a_{m}\right)=\max_{\alpha_{m}\in R}\frac{1}{2}\log^{+}\left(\frac{P_{e}}{\alpha_{m}^{2}+P_{e}{∥\alpha_{m}h_{m}-a_{m}∥}^{2}}\right)$$

where $\log^{+}\left(x\right)≜\max\left\{\log(x),0\right\}$.

To maximize $R\left(h_{m},a_{m}\right)$, the minimum value $\alpha_{m}$ can be found by setting the derivative equal to zero,

(7) $$\begin{array}{c} \frac{\partial \sigma^{2}\left(h_{m},a_{m},\alpha_{m}\right)}{\partial \alpha_{m}}=0 \end{array}$$

It is easy to find that,

(8) $$\begin{array}{cc} \frac{\partial \sigma^{2}\left(h_{m},a_{m},\alpha_{m}\right)}{\partial \alpha_{m}} & =2\alpha_{m}+2P_{e}\alpha_{m}{h_{m}}^{T}h_{m}-2P_{e}{h_{m}}^{T}a_{m} \end{array}$$

Thus, the minimum mean squared error (MMSE) coefficient $\alpha_{MMSE}$ is obtained by

(9) $$\begin{array}{c} \alpha_{MMSE}=\frac{P_{e}{h_{m}}^{T}a_{m}}{1+P_{e}{∥h_{m}∥}^{2}} \end{array}$$

We solve this $\alpha_{MMSE}$ to obtain and plug back into Equation (5). We can gain the equation as follows,

(10) $$\begin{array}{c} \sigma^{2}\left(h_{m},a_{m}\right)=P_{e}{∥a_{m}∥}^{2}-\frac{P_{e}^{2}{\left(h_{m}^{T}a_{m}\right)}^{2}}{1+P_{e}{∥h_{m}∥}^{2}} \end{array}$$

Thus, the result of the computation rate $R(h_{m},a_{m})$ of the relay is as follows ([3], Theorem 2).

(11) $$\begin{array}{c} R\left(h_{m},a_{m}\right)=\frac{1}{2}\log^{+}{\left({∥a_{m}∥}^{2}-\frac{P_{e}{\left(h_{m}^{T}a_{m}\right)}^{2}}{1+P_{e}{∥h_{m}∥}^{2}}\right)}^{-1} \end{array}$$

where $\log^{+}\left(x\right)≜\max\left\{\log(x),0\right\}$.

In addition, the ICVs $a_{m}$ must satisfy that

(12) $${∥a_{m}∥}^{2}\leq 1+P_{e}{∥h_{m}∥}^{2}$$

Therefore, it can be derived that the computation rate $R(h_{m},a_{m})\neq 0$. To ensure that the relay can correctly decade the equation, the maximum computation rate of a relay must be lower than its forwarding theoretical rate, otherwise there will be information congestion and the correct decoding will not be possible, and the relay forwarding theoretical rate is fixed $R_{mD}^{f}$. The forwarding theoretical rate is

(13) $$R_{mD}^{f}=log\left(1+\frac{P_{e}{∥f_{mD}∥}^{2}}{N_{0}}\right)$$

where $N_{0}$ is the power spectral density of the noise and $f_{mD}$ indicates the channel coefficient from relay *m* to the destination *D*.

According to Equations (11) and (13), the computation rate *R* is,

(14) $$R<\min_{m:a_{m\ell }\neq 0}\;\left(R_{mD}^{f},R\left(h_{m},a_{m}\right)\right)$$

## 4. Proposed Approach

In this section, we design a novel SECoF method based on the CoF framework by analyzing a multi-source multi-relay multi-antenna system. The main technical contribution is the generalization of the computational forwarding framework, where the relay uses multi-antennas and recovers multiple linear combinations (at a single receiver); we model our generalized framework by applying the computational forwarding framework to a classical Gaussian channel and by concisely proving it.

### 4.1. Multi-Antenna Successive Extended Computation Framework SECoF

In this subsection, we propose a novel relay forwarding SECoF method applied to multiple antennas, which utilizes the matrix projection and the SIC technique. The conventional SIC uses the previously decoded equations to improve the computational rate of the subsequent equations, which can reduce the interference and make it easier for the relay to translate the next codewords, but the SIC scheme has the problem of high complexity. Thus, to solve these problems, the SECoF utilizes a matrix projection algorithm to obtain the signal equation to reduce complexity, exploits the LLL algorithm to obtain the ICVs, and integrates parallel computation to rationalize the computation rate; we use the system model as Definition (2). The SECoF is structured into two distinct procedures: in the first procedure, we use matrix projection combined with SIC to construct an effective channel to compute the rate. In the second procedure, we use the LLL algorithm to form the ICVs.

#### 4.1.1. The Matrix Projection and SIC Based CoF Mechanism

The SECoF leverages matrix projection combined with SIC to enhance the robustness of the CoF mechanism. This combination aims to optimize signal processing, thus reducing the instances of rank failure. For this purpose, we constructed three steps. In the first step, the relay develops a multi-antenna multi-relay signaling function $\hat{F}$ by listening and detecting the previous time slots and utilizing the signals transmitted by each relay to ensure the initial value. In the second step, we construct a new effective channel removing the validity of the previous equation using the matrix projection space and obtain the computed rate $R\left(G^{m},\tau ,\theta ,\rho ,{\bar{a}}_{\zeta },P_{e}\right)$. In the third step, we calculate the minimum effective noise variance of the channel and obtain the optimal computation rate $R_{\zeta }\left(Q_{\zeta -1},{\bar{a}}_{\zeta },P_{e}\right)$.

Specifically, when the relay node is in its ζth phase, its ζth best equation will be recovered and transmitted to the destination in the corresponding time slot as follows. We assume that relay *m* has correctly decoded the first ζ−1 linear combinations and obtain the ICVs $a_{1},\ldots ,a_{\zeta -1}$ in the ζth step. For simplicity, the relay *m* will be handled by default below, omitting the subscript *m*. As with the traditional SIC method, the relay *m* has been obtained from the previous ζ−1 steps with linearly independent $D_{\zeta -1}\in R^{\left(\zeta -1\right)\times L}$, which can be presented as  

(15) $$D_{\zeta -1}={\left[a_{1},\ldots ,a_{\zeta -1}\right]}^{T}$$

In the first step, we construct the multi-antenna multi-relay signaling function $\hat{F}$, which is formulated to ensure initial values. Relay generates the following signal ${\hat{F}}_{\zeta -1}$ with the $D_{\zeta -1}$.

(16) $${\hat{F}}_{\zeta -1}=\sum_{s=1}^{\zeta -1}\sum_{\ell =1}^{L}a_{sl}x_{\ell }=D_{\zeta -1}X$$

where the $X={\left[x_{1},x_{2},\ldots x_{L}\right]}^{T}$, $X\in R^{L\times \omega }$, ${\hat{F}}_{\zeta -1}\in R^{\left(\zeta -1\right)\times \omega }$

In the second step, we obtain the computed rate $R\left(G^{m},\tau ,\theta ,\rho ,{\bar{a}}_{\zeta },P_{e}\right)$. We adopt the matrix projection space method which is different from the traditional SIC method, the correlation is removed from the received signal before successive cancellation, which can effectively reduce the complexity of the calculation. The *m*th relay attempts to recover a new equation based on the ζ−1 equations received in the previous time slots and its own received signal $y_{\zeta }^{n}$. According to the definition of projection space method [29], a vector subspace consists of projections of vectors. Since $D_{\zeta -1}$ is composed of linearly independent vectors *a*, by removing the effects of the previous equations from the received signal using the projection space method, we can obtain the projection of the matrix P,

(17) $$P=D_{\zeta -1}^{T}{\left(D_{\zeta -1}D_{\zeta -1}^{T}\right)}^{-1}D_{\zeta -1}$$

Note that the matrix $D_{\zeta -1}^{T}{\left(D_{\zeta -1}D_{\zeta -1}^{T}\right)}^{-1}D_{\zeta -1}$ is the subspace spanned by matrix $D_{\zeta -1}$, the $P\in R^{L\times L}$

Therefore, we utilize the signal received by the *n* antenna in the ζ step as $y_{\zeta }^{n}$, and the projection of ${\hat{F}}_{\zeta -1}\in R^{\left(\zeta -1\right)\times \omega }$ on the received signal $y_{\zeta }^{n}$ is removed and used to eliminate its correlation to obtain the following equation,

(18) $$\begin{array}{cc} {\hat{y}}_{\zeta }^{n} & =y_{\zeta }^{n}-g_{\zeta }^{T}PX\\ \; & =y_{\zeta }^{n}-g_{\zeta }^{T}D_{\zeta -1}^{T}{\left(D_{\zeta -1}D_{\zeta -1}^{T}\right)}^{-1}{\hat{F}}_{\zeta -1}\\ \; & =y_{\zeta }^{n}-g_{\zeta }^{T}D_{\zeta -1}^{T}{\left(D_{\zeta -1}D_{\zeta -1}^{T}\right)}^{-1}D_{\zeta -1}X \end{array}$$

where $g_{\zeta }\in R^{L}$

Then, we calculate the rate based on the new effective channel construction by using the edge information obtained from Equation (18). Operationally, the receiver forms the new effective channel, and the linear combination ${\bar{y}}_{\zeta }^{SE}$ of this signal ${\hat{y}}_{\zeta }^{n}$ and the previously derived equations ${\hat{F}}_{\zeta -1}$ is made as follows,

(19) $$\begin{array}{cc} {\bar{y}}_{\zeta }^{SE} & =\sum_{n=1}^{N}\tau_{\zeta n}{\hat{y}}_{\zeta }^{n}+\rho^{T}{\hat{F}}_{\zeta -1}\\ \; & =\sum_{n=1}^{N}\tau_{\zeta n}{\hat{y}}_{\zeta }^{n}+\rho^{T}D_{\zeta -1}X\\ \; & ={\bar{a}}_{\zeta }^{T}X+\underset{\mathrm{effective}\;\mathrm{noise}\;}{\underset{︸}{\left(\tau^{T}{\left(G^{m}\right)}^{T}\theta +\rho^{T}D_{\zeta -1}-{\bar{a}}_{\zeta }^{T}\right)X+\tau^{T}Z^{m}}} \end{array}$$

where $G^{m}\in R^{L\times N}$, $Z^{m}\in R^{N\times \omega }$

(20) $$\rho ={\left[\rho_{1},\rho_{2},\ldots ,\rho_{\zeta -1}\right]}^{T},\rho \in R^{\zeta -1},$$

(21) $$\tau ={\left[\tau_{\zeta 1},\ldots ,\tau_{\zeta N}\right]}^{T},\tau \in R^{N},$$

(22) $$\theta ≜\left(I-D_{\zeta -1}^{T}{\left(D_{\zeta -1}D_{\zeta -1}^{T}\right)}^{-1}D_{\zeta -1}\right)$$

and $\theta \in R^{L\times L}$.

Hence, the computation rate of an equation with ICVs ${\bar{a}}_{\zeta }$ is

(23) $$\begin{array}{cc} \; & R\left(G^{m},\tau ,\theta ,\rho ,{\bar{a}}_{\zeta },P_{e}\right)\\ \; & =\frac{1}{2}\log^{+}\left(\frac{P_{e}}{{∥\tau ∥}^{2}+{∥\left(\tau^{T}{\left(G^{m}\right)}^{T}\theta +\rho^{T}D_{\zeta -1}-{\bar{a}}_{\zeta }^{T}\right)P_{e}^{\frac{1}{2}}∥}^{2}}\right) \end{array}$$

where the $\log^{+}\left(x\right)≜\max\left\{\log(x),0\right\}$.

In the third step, to maximize the computation rate $R\left(G^{m},\tau ,\theta ,\rho ,{\bar{a}}_{\zeta },P_{e}\right)$ in Equation (23), the optimal values of τ and ρ and the corresponding maximum computation rate $R_{\zeta }\left(Q_{\zeta -1},{\bar{a}}_{\zeta },P_{e}\right)$ can be determined according to the following Theorem 1. We calculate the minimum effective noise variance of the channel and obtain the optimal computation rate according to Equation (23). It is worth noting that the magnitudes of τ and ρ are found to be negatively related to $R\left(G^{m},\tau ,\theta ,\rho ,{\bar{a}}_{\zeta },P_{e}\right)$ by Equations (A3) and (A4), and therefore, by adjusting the coefficient vectors τ and ρ to improve the relay computation rate. In summary, we obtain the optimal computation speed, and the specific result is shown in Theorem 1.

**Theorem** **1 (SECoF method).** *For a wireless relay network, which has L transmitters, M relay nodes, each with N antennas, energy limit $P_{e}$, and one destination node, supposing rank of $D_{\zeta -1}=\zeta -1$, and the $D_{0}=0$, the optimal computation rate accessible to the relay m is:*

(24) $$\begin{array}{cc} \; & R_{\zeta }\left(Q_{\zeta -1},{\bar{a}}_{\zeta },P_{e}\right)\\ \; & =\frac{1}{2}\log^{+}\left(\frac{P_{e}}{{\bar{a}}_{\zeta }^{T}Q_{\zeta -1}{\bar{a}}_{\zeta }}\right) \end{array}$$

*where $P_{e}=diag\left(P_{e}\right)$, and $P_{e}\in R^{L\times L}$,*

(25) $$\begin{array}{cc} \; & Q_{\zeta -1}≜\left(I-\left(TD_{\zeta -1}^{T}\right){\left(D_{\zeta -1}TD_{\zeta -1}^{T}\right)}^{-1}D_{\zeta -1}\right)\left(I-D_{\zeta -1}^{T}{\left(D_{\zeta -1}TD_{\zeta -1}^{T}\right)}^{-1}\left(D_{\zeta -1}T\right)\right), \end{array}$$

*and $Q_{\zeta -1}\in R^{L\times L}$,*

(26) $$T≜P_{e}-\theta P_{e}G^{m}{\left(I+{\left(G^{m}\right)}^{T}\theta P_{e}\theta^{T}G^{m}\right)}^{-1}{\left(G^{m}\right)}^{T}P_{e}\theta ,andT\in R^{L\times L}$$

The proof can be found in Appendix A.

#### 4.1.2. LLL Algorithm to Find the Suboptimal ICVs

The LLL algorithm, originally designed for mesh reduction, is reused in this context to simplify the ICV process. According to the Theorem 1, the matrix $Q_{\zeta -1}$ and the LLL algorithm need to be used to find the suboptimal ICVs $a_{\zeta }$ in order to maximize the computation rate.

**Theorem** **2 (Symmetry matrix).** *Given a square matrix A, where A is an n×n matrix, we can assert that A is a symmetric matrix if and only if it satisfies the following condition:*

$A=A^{T}$

**Theorem** **3 (Positive Definite Matric).** *Given a square matrix A, where A is an n×n matrix, we can assert that A is a positive definite matrix if and only if it satisfies the following conditions:*

*(1) Symmetry: Matrix A must be a symmetric matrix, meaning that $A=A^{T}$, where $A^{T}$ represents the transpose of matrix A.*

*(2) Positive definiteness: For all nonzero column vectors x (x≠0), the following condition must hold:*

$x^{T}Ax>0$

*Here, $x^{T}$ denotes the transpose of vector x.*

The proof of Theorem 3 follows.

**Proof.**  First, the proof is carried out for the symmetry of the matrix *A*

If matrices $A=A^{T}$ and *A* are square matrices, then matrix *A* is a symmetric matrix.

Second, we prove the positive characterization of *A*.

Assuming that the eigenvalue of A is λ, according to the eigenvalue, the formula is

(27) $$\begin{array}{cc} \; & Ax=\lambda x\left(x\neq 0\right)\\ \; & \Rightarrow x^{T}Ax=x^{T}\lambda x=\lambda {∥x∥}^{2} \end{array}$$

According to $x^{T}Ax>0$, the $\lambda {∥x∥}^{2}>0x\neq 0$, and, we can find the eigenvalue as follows,

(28) λ>0

Therefore, we can obtain the matrix *A* as a positive definite matrix.    □

**Lemma** **1.** *Assuming that the vector $a_{\zeta }\neq 0$, the $Q_{\zeta -1}$ is the symmetric and positive definite matrix.*

**Proof.**  (1) We prove $Q_{\zeta -1}$ is the symmetry matrix according to the Theorem 2.

Firstly, it follows from Equation (25) that $Q_{\zeta -1}$ is a square matrix, and the size of $Q_{\zeta -1}$ is L×L.

Secondly, according to the sufficient condition for the symmetry matrix from Theorem 3, we only need to prove that $Q_{\zeta -1}=Q_{\zeta -1}^{T}$. The transpose matrix $Q_{\zeta -1}^{T}$ is as follows,

(29) $$\begin{array}{cc} \; & {Q_{\zeta -1}}^{T}≜{\left(\left(I-\left(TD_{\zeta -1}^{T}\right){\left(D_{\zeta -1}TD_{\zeta -1}^{T}\right)}^{-1}D_{\zeta -1}\right)\left(I-D_{\zeta -1}^{T}{\left(D_{\zeta -1}TD_{\zeta -1}^{T}\right)}^{-1}\left(D_{\zeta -1}T\right)\right)\right)}^{T}\\ \; & \;\;\;\;\;\;\;=\;\left(I^{T}-{\left(D_{\zeta -1}T\right)}^{T}{\left(D_{\zeta -1}TD_{\zeta -1}^{T}\right)}^{-1}D_{\zeta -1}\right)\left(I^{T}-D_{\zeta -1}^{T}{\left(D_{\zeta -1}TD_{\zeta -1}^{T}\right)}^{-1}{\left(TD_{\zeta -1}^{T}\right)}^{T}\right)\\ \; & \;\;\;\;\;\;\;=\left(I-\left(TD_{\zeta -1}^{T}\right){\left(D_{\zeta -1}TD_{\zeta -1}^{T}\right)}^{-1}D_{\zeta -1}\right)\left(I-D_{\zeta -1}^{T}{\left(D_{\zeta -1}TD_{\zeta -1}^{T}\right)}^{-1}\left(D_{\zeta -1}T\right)\right)\\ \; & \;\;\;\;\;\;\;=Q_{\zeta -1} \end{array}$$

Therefore, the ${Q_{\zeta -1}}^{T}$ is the symmetric matrix.

(2) The following step is to proof the ${Q_{\zeta -1}}^{T}$ is the positive definite matrix according to Theorem 3. We can find the following:

(30) $$\begin{array}{cc} \; & Q_{\zeta -1}≜\left(I-\left(TD_{\zeta -1}^{T}\right){\left(D_{\zeta -1}TD_{\zeta -1}^{T}\right)}^{-1}D_{\zeta -1}\right)\left(I-D_{\zeta -1}^{T}{\left(D_{\zeta -1}TD_{\zeta -1}^{T}\right)}^{-1}\left(D_{\zeta -1}T\right)\right)\\ \; & \;\;\;\;\;\;=VV^{T} \end{array}$$

where the $V=I-\left(TD_{\zeta -1}^{T}\right){\left(D_{\zeta -1}TD_{\zeta -1}^{T}\right)}^{-1}D_{\zeta -1}$, and $V\in R^{L\times L}$

Therefore, we will argue that the effective noise variance is

(31) $$\begin{array}{cc} \; & \sigma_{SECoF}^{2}\left(T,P_{e},G^{m},\theta \right)={\bar{a}}_{\zeta }^{T}Q_{\zeta -1}{\bar{a}}_{\zeta }^{}\\ \; & \;\;\;\;\;\;\;\;\;\;\;\;\;\;\;\;={\bar{a}}_{\zeta }^{T}VV^{T}{\bar{a}}_{\zeta }^{}\\ \; & \;\;\;\;\;\;\;\;\;\;\;\;\;\;\;\;={∥V{\bar{a}}_{\zeta }^{}∥}^{2} \end{array}$$

Note that ${\bar{a}}_{\zeta }^{}\neq 0$, so $\sigma_{SECoF}^{2}\left(T,P_{e},G^{m},\theta \right)>0$.

According to Theorem 3, we assume that the eigenvalue of ${Q_{\zeta -1}}^{T}$ is λ; according to the eigenvalue, the formula is

(32) $$\begin{array}{cc} \; & Q_{\zeta -1}{\bar{a}}_{\zeta }=\lambda {\bar{a}}_{\zeta }\\ \; & \Leftrightarrow {\bar{a}}_{\zeta }^{T}Q_{\zeta -1}{\bar{a}}_{\zeta }={\bar{a}}_{\zeta }^{T}\lambda {\bar{a}}_{\zeta }=\lambda {∥{\bar{a}}_{\zeta }∥}^{2} \end{array}$$

Due to ${\bar{a}}_{\zeta }^{}\neq 0$ and ${\bar{a}}_{\zeta }^{T}Q_{\zeta -1}{\bar{a}}_{\zeta }>0$, we can obtain that $\lambda {∥{\bar{a}}_{\zeta }∥}^{2}>0$

Therefore, the λ>0 and the matrix $Q_{\zeta -1}$ is a positively definite matrix.

So, it is clear that $Q_{\zeta -1}$ is a symmetric and positive definite matrix.    □

According to the Lemma 1, we can use the LLL algorithm to realize the construction of suboptimal ICVs. The LLL algorithm is an algorithm for performing lattice reduction on a given set of vectors. Its main goal is to transform a set of linearly correlated vectors into nearly linearly uncorrelated vectors. The output of the LLL algorithm is still a linear combination of the original vectors, so it does not completely eliminate linear correlation. The LLL algorithm may produce good results, making the vectors nearly linearly uncorrelated. Since it is very difficult to obtain ICVs in the CoF, we can calculate a set of nearly linearly uncorrelated vectors by using the LLL algorithm, choose the row of linearly uncorrelated vectors with the smallest norm among them, and finally construct the full-rank ICV matrix A.

We now give more details on determining A using the LLL algorithm. We determine A in a column-by-column manner from $a_{1}$ to $a_{L}$. For any step ζ and the ζ∈L, since the $Q_{\zeta -1}$ is a symmetric positive definite matrix, it is possible to perform a Cholesky decomposition of $Q_{\zeta -1}$. First, we utilize the Cholesky decomposition of $Q_{\zeta -1}$, that is,

(33) $$Q_{\zeta -1}=L_{\zeta }L_{\zeta }^{T}$$

where the $Q_{\zeta -1}$ is the Gram matrix for a lattice with generator matrix $L_{\zeta }$

Second, apply the LLL algorithm to $L_{\zeta }$ to find the reduced matrix $L_{\zeta }^{{}^{\prime }}$.

In addition, we compute the $V_{\zeta }$,

(34) $$V_{\zeta }=L_{\zeta }^{{}^{\prime }}L_{\zeta }^{-1}$$

Finally, we choose the $a_{\zeta }$ as the rows of $V_{\zeta }$ to obtain the smallest norm which is linearly independent of $a_{1},\ldots ,a_{\zeta -1}$. The suboptimal ICV A is,

(35) $$A=\left[a_{1},\ldots ,a_{\zeta -1},a_{\zeta }\right]$$

Therefore, we can obtain coefficient $a_{\zeta }$ in the step ζ, and the matrix A is a full rank.

The computational range proposed in this paper can be applied to multi-antenna relays. By adapting the coefficients τ and ρ, we can make it easier for the relay to decode the received linear combinations and can effectively improve the range of computation rates of relays.

### 4.2. SECoF Method

This section describes how the SECoF computing scheme is implemented. The SECoF computing scheme is divided into four main processes, and the pseudo-code of the algorithm is shown in Algorithm 1.
**Algorithm 1:** Pseudocode of SECoF scheme
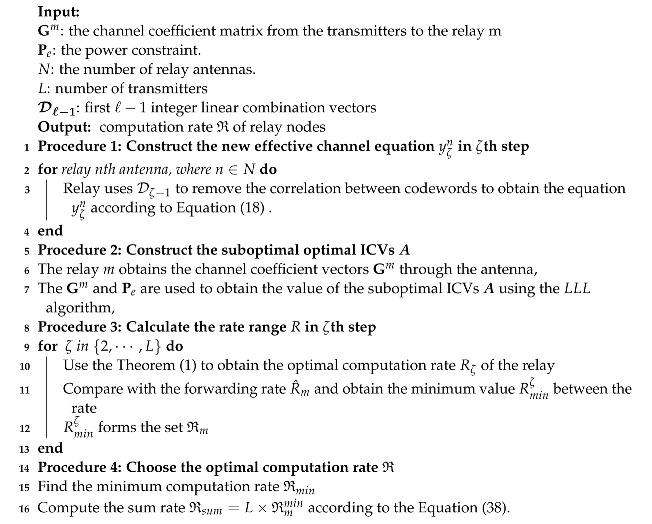


Procedure 1: Removal of correlation between received signals. The relay *m* has been obtained the linearly independent matrix $D_{\zeta -1}$, and generates the signal as Equation (18). Then, we adopted the matrix projection method to construct a new effective equation to remove the correlation from the received signal $y_{\zeta }^{n}$.

Procedure 2: In the ζth step, according to the fact that $Q_{\zeta -1}$ is a symmetric and positive definite matrix, the relay *m* obtains the suboptimal ICVs $a_{\zeta }$ by using the LLL algorithm for the multi-antenna channel matrix $Q_{\zeta -1}$.

Procedure 3: Construct a new effective equation. According to the SIC scheme, the relay forms a new effective channel observation equation Equation (19), the current equation is decoded by using the previous equation, and the computation rate is calculated using Equation (24).

Procedure 4: Choose the optimal computation rate. We can obtain the rate tuple $U_{rate}=\left\{R_{1},\ldots ,R_{L}\right\}$, and the relay forwarding rate according to the channel is,

(36) $$\begin{array}{c} {\hat{R}}_{m}=\log\left(1+\frac{P_{e}}{N}{\left|h_{f}\right|}^{2}\right) \end{array}$$

where the $\log^{+}\left(x\right)≜\max\left\{\log(x),0\right\}$,the $h_{f}$ is the channel coefficient of the relay to the destination node.

According to the CoF scheme, we should find the minimum computation rate, and the computation rate of the relay must be lower than the relay forwarding rate; otherwise, the destination node cannot receive L-independent equations and generate a loss, so the actual rate $R_{m}$ is

(37) $$\begin{array}{c} R_{m}=min\left({\hat{R}}_{m},U_{rate}^{min}\right) \end{array}$$

As a result, the sum rate of recovering all messages from these *L* equations is as follows,

(38) $$\begin{array}{c} R_{sum}=R_{m}\times L \end{array}$$

## 5. Performance Evalution

In this section, we provide computer simulation experiments with different numbers of transmitters, relays, antennas, and a different SNR and outage probability to illustrate the network performance metrics of the proposed schemes SECoF. The simulation present random channel coefficient vectors $g_{ln}^{m}∼N\left(0,1\right)$ between the *ℓ*th transmitter and *n*th antenna of the receiver *m* and the power constraints $P_{e}$ in ideal relay-to-destination channels. We search for the optimal ICVs matrix A by using the LLL algorithm. For each experiment, randomly generated the value of $G^{m}$ 10,000 times and calculated the average sum rates to make the experiment more general.

We compare the following seven relaying methods with different networks, and briefly recall their working principles.

(1) AF [7]: Amplify-and-Forward is a relay communication strategy in which the relay node receives the signal from the source node, amplifies it, then forwards it to the destination node. This method is commonly used in wireless communications where the relay node does not perform decoding operations.
(2) DF [9]: Decode-and-Forward is a relay communication strategy in which a relay node receives a signal from a source node, attempts to decode it, then re-encodes and transmits it to the destination node. This method is often used to improve network performance and reduce transmission errors.
(3) Orig.CoF [3]: A Compute-and-Forward strategy in which relay nodes perform computation operations and then transmit the computation results to the target node.
(4) SucCoF [30]: Successive CoF is the relay node that can continuously perform multiple calculation operations and then transmit these calculation results to the target node to further improve network performance.
(5) SECoF: Successive extended CoF, the algorithm proposed in this paper.
(6) Cut-set bound [28]: The cut-set bound limit is a concept used to measure network capacity and performance. It represents the maximum number of possible cut-sets in the network, a cut-set is a set of nodes or edges whose removal will cause the network to separate. Cut-set bound can be used to evaluate the capacity limits of a network.

### 5.1. Complexity Analysis

In this subsection, the complexity of SECoF is analyzed. In this algorithm, *N* denotes the number of antennas, *L* denotes the number of transmitter nodes and*M* denotes the number of relay nodes. With Algorithm 1, it is easy to find its complexity is o(LNM).

### 5.2. Rank Failure Probability

**Figure 2:**
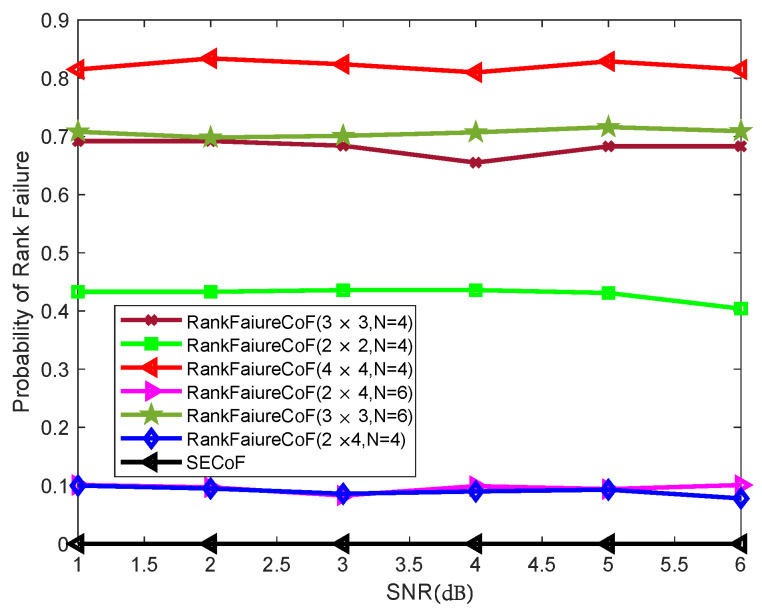
The performance rank failure of various relaying schemes.

In this subsection, we illustrate the probability of rank failure at the destination for Orig.CoF and SECoF in Figure 2. The probability of rank failure generated by SECoF methods is zero because *L* linear independent equations are sent from the relay to the destination node by using extended computation and SIC methods. However, in the Orig.CoF method, the relay can send the equation independently, so Orig.CoF is prone to linear correlation of equations and rank failure. It can be seen that as the number of relays increases, the probability of rank failure gradually decreases, the number of received equations of the destination node increases, and the probability of finding *L* linear independent linear uncorrelated equations is higher. In addition, as the number of transmitters *ℓ* increases, the probability of rank failure gradually increases because the relay receives more equations, but the probability of phase dependence between equations also increases, and the equations cannot be reasonably recovered. It can be seen from the figure that as the antenna *N* increases, the relay has a relatively small impact on the rank failure probability because the relay transmits the information to the destination node using one antenna. Therefore, the SECoF methods have significant advantages in compensating for the rank failure.

### 5.3. Comparison of Various Relaying Schemes

**Figure 3:**
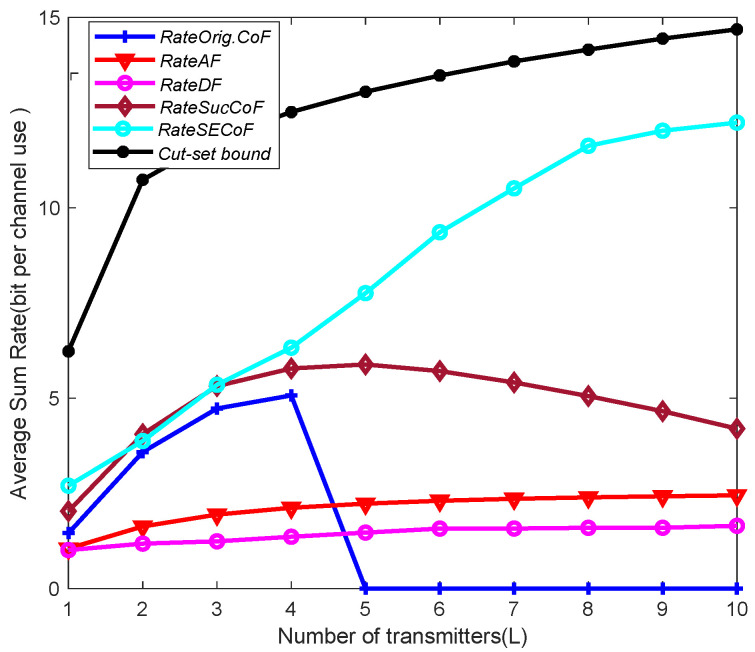
Average sum rate versus the number of transmitters L for SNR = 10, M = 4, and N = 3.

Figure 3 shows the effect of the number of transmitters, L, on the network performance for different forwarding schemes. The figure shows the relationship between the average sum rate of Orig.CoF, SucCoF, cut-set bound, SECoF, AF, DF and the number of transmitters *ℓ* when SNR = 10 dB, M = 4 and N = 3. It can be seen from the figure that as *ℓ* increases, the trend of the average sum rates increases. In addition, the SECoF method outperforms the other schemes and the performance gap continues to increase. The reason is that SECoF uses matrix projection based on SIC to reduce the algorithm complexity and improve the network performance. Therefore, it can be applied in a network environment with a large number of transmitters. On the other hand, Orig.CoF only implements forwarding according to the number of equations currently received, but it suffers from the rank failure problem and rate loss. The AF method and the DF method cannot handle the problem of increasing interference in the signal as the number of *L* increases. Therefore, the SECoF proposed in this paper effectively improves the performance of the network.

**Figure 4:**
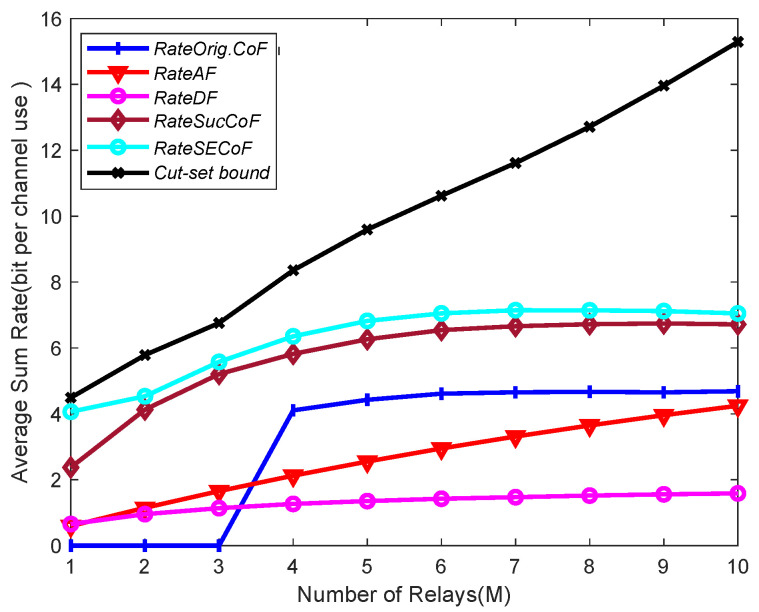
Average sum rate versus the number of relays M for SNR = 10, L = 4, and N = 3.

In Figure 4, we illustrate the impact of using a large number of relays *M* on network performance by comparing the average sum rates of Orig.CoF, SucCoF, cut-set bound, SECoF, AF, and DF for different numbers of relays *M* at SNR = 10 dB, N = 4, and L = 3. As we can see from the figure, the trend of the average sum rates increases with the increase in *M*. By increasing the number of relays *M*, more rate options can be obtained for the destination node. The probability of rank failure of Orig.CoF can be effectively reduced by increasing the number of relays *M*, so the destination node can have a higher chance of decoding, which effectively improves the computation rate. By increasing the number of relays *M*, the number of antennas that can be received also increases, and the destination node can receive more equations for recovering equations at a higher rate, so as to achieve a wider rate. With the increase in the number of relays *M*, the number of recovery equations available to the destination node in SECoF also increases, which can effectively prevent the rank failure problem prone to Orig.CoF.

**Figure 5:**
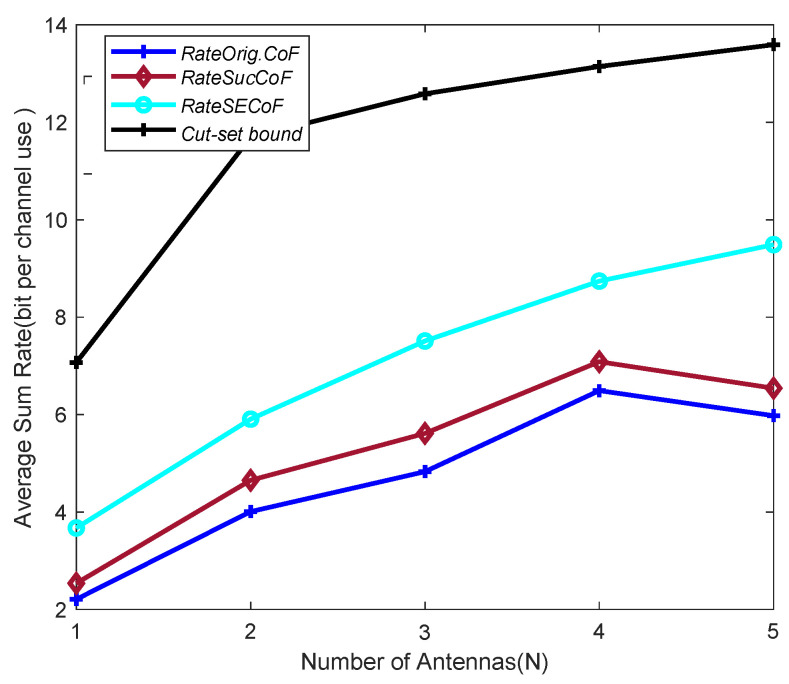
Average sum rate versus the number of antennas N for SNR = 10, M = 4, and L = 4.

It is interesting to compare the effects of the Orig.CoF SECoF methods on the network performance for relays with different numbers of antennas *N* at SNR = 10 dB, M = 4 and L = 4, as shown in Figure 5. It can be seen from the figure that the trend of the average sum rate increases with the number of antennas *N*. The relays can receive more signals by increasing the number of antennas and combining them to obtain a more efficient rate recovery equation. The average sum rate obtained by the Orig.CoF is the lowest, and the multiple antennas have little effect on it and are more prone to produce rank failures. In contrast, the SECoF proposed in this paper increases the recoverable equation because of the increase in the number of antennas, which improves the efficiency of the relay translation correlation equation. Thus, the SECoF can obtain a more effective equation for rate improvement.

**Figure 6:**
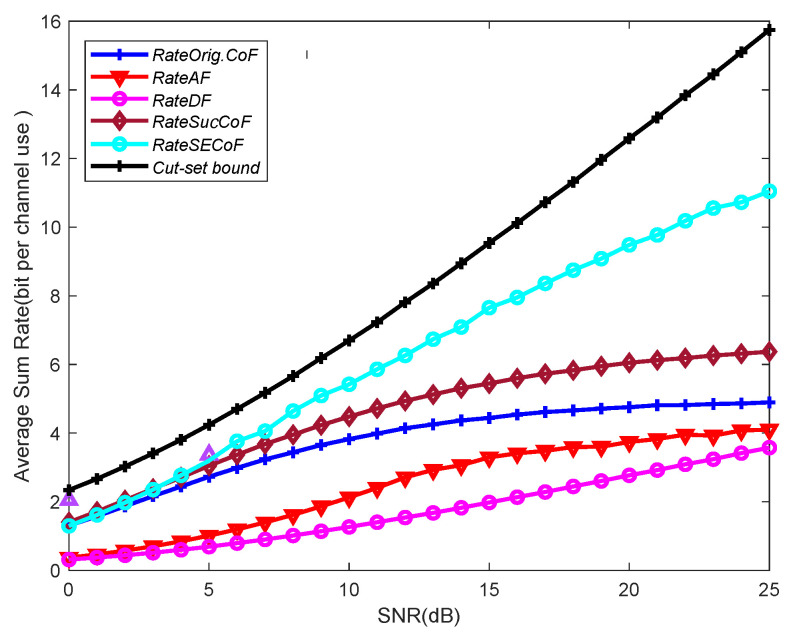
Average sum rate versus the SNR for L = 4, M = 4, and N = 3.

In Figure 6, we illustrate the comparison of the network performance of the Orig.CoF, SucCoF, cut-set bound, SECoF, AF, and DF methods under different SNRs. We observe that the SECoF method performs better than the other relay forwarding methods, and the DF method has the lowest average sum rate among all forwarding methods because the DF method needs to decode packets one by one and then forward them again, which is prone to packet congestion problems. The CoF-based relay forwarding method has a higher rate than AF and DF because the CoF strategies use interference to achieve higher computation rates, while DF and AF have interference-limited relays as interference increases. SECoF has more versatility and can achieve a wider rate range. Therefore, SECoF has better network performance.

### 5.4. Comparison in Various Network Sizes

In this subsection, we compare the network performance of Orig.CoF, SECoF, AF, and DF by comparing the cases of different network sizes. By comparing different network sizes, it is possible to more intuitively observe the impact of different algorithms on general and representative network performance. We set up two different network sizes as follows,

(1) Wireless relay network with sizes 4×4.
(2) Wireless relay network with sizes 2×2.

**Figure 7:**
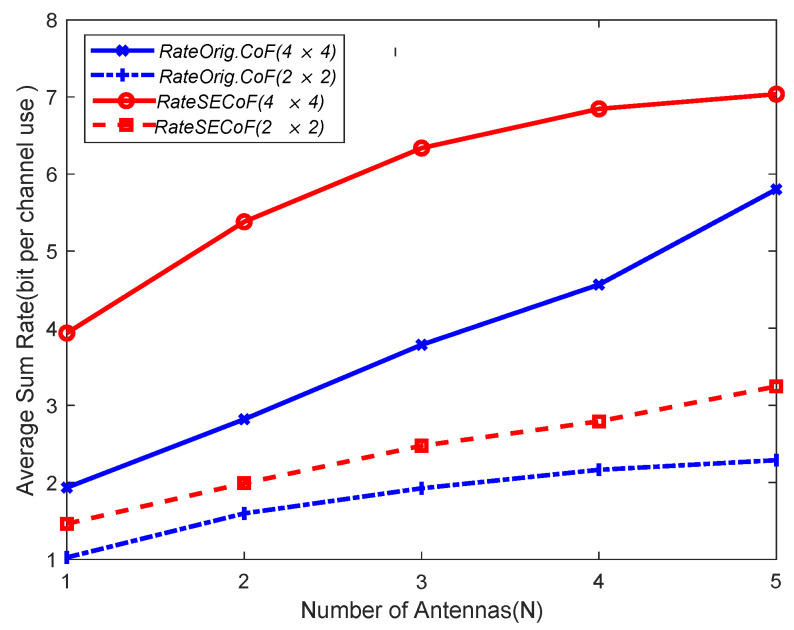
The performance comparison of various schemes versus number of antennas N for relay network with different network sizes.

As shown in Figure 7, the network performance of SECoF and Orig.CoF is compared with the different number of antennas under the above network size. The result shows that SECoF has the best network performance and Orig.CoF has the worst network performance. Furthermore, the performance of the two methods is gradually improved as the network size increases. For example, in the network size of 2×2, the computation rate is the lowest because Orig.CoF transmission cannot overcome the rank failure problem. But SECoF can handle the rank failure problem and therefore has a higher rate. As the number of antennas increases, the number of equations received by SECoF and Orig.CoF also increases, which improves the robustness of the network and reduces the linear correlation of the received equations.

**Figure 8:**
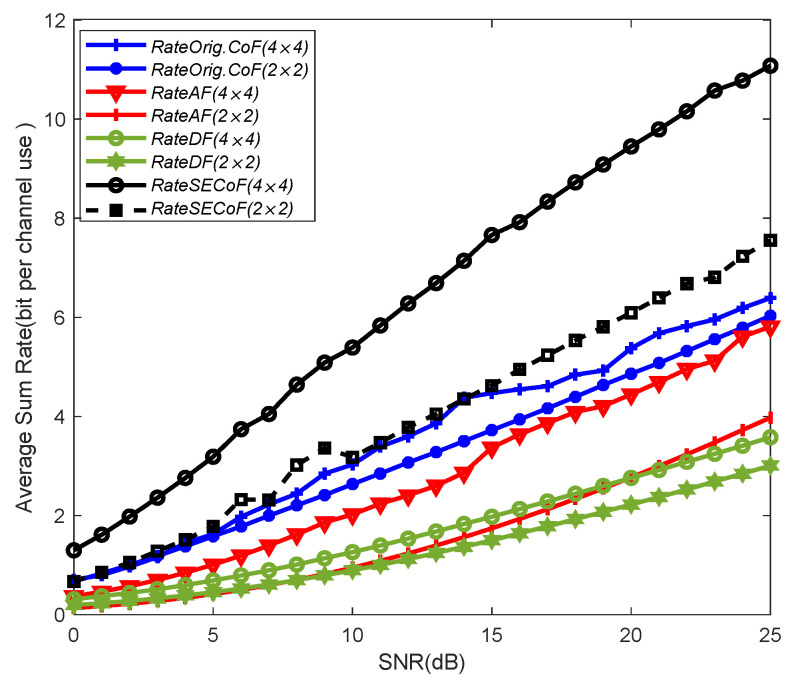
The performance comparison of various schemes versus SNR for relay network with different network sizes.

As shown in Figure 8, the network performance of Orig.CoF, AF, DF and SECoF in the above network sizes with different SNRs are compared. As observed in the figure, we can see that SECoF has the best network performance and DF has the worst network performance. The reason is that SECoF methods use the CoF framework to achieve joint decoding of signals instead of individual decoding and forwarding, and effectively overcome the rank failure problem, which helps to improve the network performance. With the increase in the network size, the network performance is improved to some extent, because with the increase in the number of transmitters and receivers, the probability of rank failure in the network decreases, and the number of received signals increases. Therefore, the overall network performance can be effectively improved by the algorithm proposed in this paper.

### 5.5. Network Outage Probabilities

In this subsection, the loss probability of the network is analyzed in two aspects. Firstly, the network performance is discussed for different SNRs in a 2×2 network, and secondly, a full comparative analysis is performed for a different number of antennas and SNRs for a network size of 4×4 and 2×2.

**Figure 9:**
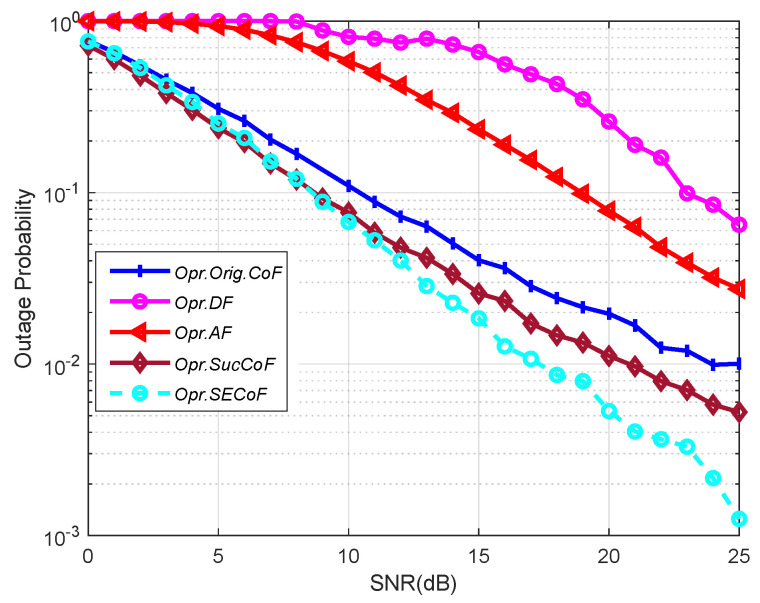
The performance comparison of various schemes versus number of SNR for L = 4, M = 4, N = 3, and $R_{t}=1$.

As shown in Figure 9, by comparing the outage probability ratios of Orig.CoF, AF, DF, and SECoF under different SNR, the threshold value $R_{t}$ in this experiment is 1. We find that DF has the highest outage probability because DF is forwarded by decoding and forwarding simultaneously, resulting in the inability to process more data at the same time, leading to a lower computation rate, and thus the relay cannot receive the relevant data. Due to the rank failure problem in Orig.CoF, some messages are linearly correlated and cannot be processed correctly, resulting in a relay computation rate below the threshold. As a result, SECoF has lower reception loss rates and better network performance.

**Figure 10:**
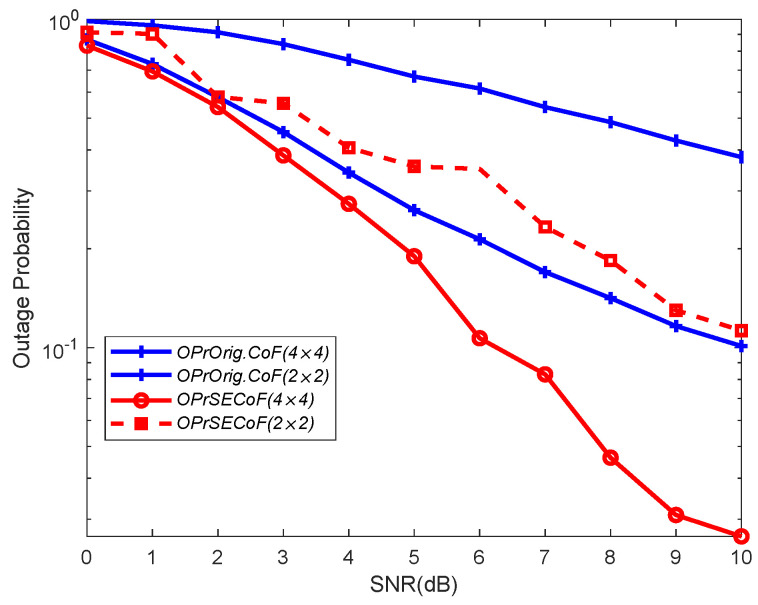
Outage probabilities of the different methods versus average SNR with different network sizes ($R_{t}=2$).

**Figure 11:**
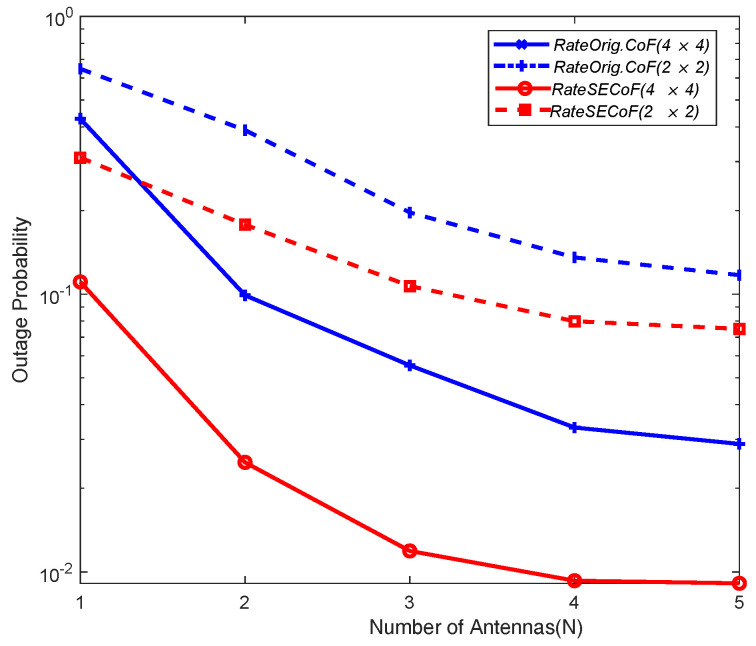
Outage probabilities of the different methods versus number of antennas N with different network sizes ($R_{t}=2$).

In Figure 10 and Figure 11, we compare the outage probability between Orig.CoF and SECoF for different network sizes, considering different numbers of antennas (N) and SNRs, respectively. Clearly, Orig.CoF consistently exhibits a higher outage probability, while SECoF consistently exhibits the lowest of the two configurations for both network sizes considered. Although increasing the number of antennas can increase the amount of received signals and improve the SNR, it does not fundamentally address the problem of rank failure. As a result, the failure probability of SECoF is minimized, demonstrating its optimal performance.

## 6. Conclusions

In this paper, we propose a new method for CoF schemes based on SECoF for multi-antenna relay networks. We concisely formulate the SECoF method, both by utilizing the LLL algorithm to find the suboptimal ICVs in the method framework and concisely deriving the corresponding formulation framework. The method is not only applicable to an arbitrary number of transmitting and receiving nodes and can solve the case where the relay contains multiple antennas, but also improves the computation rate of the relay and solves the rank failure problem. Compared with the traditional CoF, SECoF overcomes the rank failure problem and effectively improves the computation rate. Experimental results show that the network performance of SECoF proposed in this paper is significantly better than other relaying methods in terms of average sum rate and outage probability.

The SECoF has some drawbacks that need to be improved. The method needs to expand the range of applicable channels since SECoF is only applicable to real-valued channels, and future research will focus on the expansion of the methods to complex-valued signals. In addition, the methods need to be further simplified, the intermediate framework is still cumbersome, the computational efficiency needs to be improved and the overall system design needs to be carried out rather than just focusing on improving the computation rate of this point.

In the future, we will research the following aspects. Firstly, since the channel model is a real-valued channel in this paper, the complex-valued channel model can be studied in the future to make it more general. Secondly, further improvements can be made to the framework of CoF to further reduce redundant information.

## Figures and Tables

**Table 1:** List of related work.

Author	Problem	Characteristics
Shmuel, et al. [15]	Forwarding strategy	New scheduling mechanism
	optimization problem	for transmission nodes
Jeyalakshmi, et al. [16]	Forwarding strategy	Vector quantization and
	optimization problem	computational forwarding
		relaying technique
Azimi-Abarghouyi, et al. [17]	Forwarding strategy	A discrete computational
	optimization problem	forwarding scheme
Hejazi, M, et al. [18]	Forwarding strategy	Using successive interference cancelation
	optimization problem	to enhance system performance
Insausti, X, et al. [19]	Forwarding strategy	New slow block fading Gaussian
	optimization problem	access relay channel scheme
Ngeth, R, et al. [20]	Forwarding strategy	Utilizes the chunking idea
	optimization problem	and uses random linear packet codes
Zhou, B, et al. [21]	Forwarding strategy	Convert real-valued approximations
	optimization problem	to the desired set of integer-valued vectors
Sahraei, S, et al. [22]	Shortest vector	A new lattice coding to
	for the design problem	solve the shortest vector problem
Huang, Q, et al. [23]	Shortest vector	Exhaustive search and selection factors
	for the design problem	to solve the shortest vector problem
Sahraei, et al. [24]	Shortest vector	An exact polynomial complexity
	for the design problem	reduction algorithm
Wen J, et al. [25]	Shortest vector	A Linearithmic Time Algorithm
	for the design problem	for a Shortest Vector Problem
Jeyalakshmi, et al. [26]	Shortest vector	An optimal coefficient selection algorithm
	for the design problem	for a Shortest Vector Problem
Tan, Y., et al. [27]	Optimization for	A computationally compressed
	CoF framework problem	forwarding framework
Cheng, H, et al. [28]	Optimization for	A general compression framework
	CoF framework problem	

## Data Availability

No data was used for the research described in the article.

## Abbreviations

**Acronym**	**Definition**
CoF	Compute-and-Forward
WSNs	Wireless sensor networks
ICVs	Integer value coefficient vectors
AF	Amplify-and-Forward
DF	Decode-and-Forward
SIC	Successive interference cancellation
LLL	Lenstra–Lenstra–$Lov\overset{´}{a}sz$
SNR	Signal-to-noise ratio
AWGN	Additive White Gaussian Noise
NOMA	Non-Orthogonal Multiple Access
DCMF	Discrete computational forwarding
Ext-CM	Extended computing
Suc-CM	Successive computing
OCC	Overlapping chunked code
CCF	Compressed forwarding framework
GCCF	General compression framework
CSI	Channel state information
MMSE	Minimum mean squared error
Orig.CoF	Original CoF
SucCoF	Successive CoF
SECoF	Successive extended CoF
**Notation**	**Definition**
$x_{\ell }$	Transmitted signal of the *ℓ*th transmitter
$w_{\ell }$	The message of the *ℓ*th transmitter
$h_{m\ell }$	Real-valued channel gain
$z_{m}$	The noise vector received by relay *m*
$h_{m}$	*m*th channel vector
$z_{n}^{m}$	The noise vector received by *n*th antenna relay *m*
H	The channel matrix
$g_{\ell n}^{m}$	The signal channel coefficient received by the *n*th antenna of relay *m* from transmitter *ℓ*
$g^{m}$	The set of channel coefficients of the signal received by one antenna of relay *m*
$G^{m}$	The signal channel coefficients matrix received by the relay *m*
$u_{m}$	A linear combination of messages $w_{\ell }$
a	The set of integer value coefficient vectors
R	Computation rate region
$P_{e}$	Power constraint
$R_{mD}^{f}$	The relay forwarding theoretical rate
$f_{mD}$	The channel coefficient from relay *m* to the destination *D*
$D_{\zeta -1}$	The relay *m* obtains from the previous ζ−1 ICVs

## Appendix A. Proof of Theorem 1

We treat the two terms above separately. In the first step, the optimal $\tau^{opt}$ and $\rho^{opt}$ are found, respectively, based on the received equations. In the second step, $\tau^{opt}$ and $\rho^{opt}$ are brought into the denominator of the computation rate. The $\tau^{opt}$ and $\rho^{opt}$ stands for the variable coefficient under the normalized equation.

**Proof.** According to the (24), the efficient noise is

(A1) $$\begin{array}{cc} \; & \sigma_{SE}^{2}\left(G^{m},\tau ,{\bar{a}}_{\zeta },\theta ,\rho ,P_{e}|D_{\zeta -1}\right)\\ \; & ≜{∥\tau ∥}^{2}+{∥\left(\tau^{T}{\left(G^{m}\right)}^{T}\theta +\rho^{T}D_{\zeta -1}-{\bar{a}}_{\zeta }^{T}\right)P_{e}^{\frac{1}{2}}∥}^{2} \end{array}$$

Thus, the computation rate is

(A2) $$\begin{array}{cc} \; & R\left(G^{m},\tau ,\rho_{\zeta },{\bar{a}}_{\zeta },P_{e}\right)\\ \; & =\frac{1}{2}\log^{+}\left(\frac{P_{e}}{{∥\tau ∥}^{2}+{∥\left(\tau^{T}{\left(G^{m}\right)}^{T}\theta +\rho^{T}D_{\zeta -1}-{\bar{a}}_{\zeta }^{T}\right)P_{e}^{\frac{1}{2}}∥}^{2}}\right) \end{array}$$

Suppose that given ${\bar{a}}_{\zeta }$, the computation rate can be maximized by optimizing $\tau^{opt}$ and $\rho^{opt}$,

(A3) $${\rho_{\zeta }^{opt}}^{T}=\left({\bar{a}}_{\zeta }^{T}TD_{\zeta -1}^{T}\right){\left(D_{\zeta -1}TD_{\zeta -1}^{T}\right)}^{-1}$$

(A4) $$\begin{array}{c} \tau^{opt}={\left(I+{\left(G^{m}\right)}^{T}\theta P_{e}\theta^{T}G^{m}\right)}^{-1}{\left(G^{m}\right)}^{T}P_{e}\theta \left({\bar{a}}_{\zeta }-D_{\zeta -1}^{T}\rho \right) \end{array}$$

where $T=P_{e}-\theta P_{e}G^{m}{\left(I+{\left(G^{m}\right)}^{T}\theta P_{e}\theta G^{m}\right)}^{-1}{\left(G^{m}\right)}^{T}P_{e}\theta$, and $T\in R^{L\times L}$ The proof can be found in Appendix B. Hence, the computation rate of ${\bar{a}}_{\zeta }^{T}$ from (A2) is

(A5) $$R_{max}^{\zeta }=\frac{1}{2}\log^{+}\left(\frac{P_{e}}{\left({\bar{a}}_{\zeta }^{T}Q_{\zeta -1}{\bar{a}}_{\zeta }\right)}\right)$$

where $Q_{\zeta -1}\in R^{L\times L}$, and

(A6) $$\begin{array}{cc} \; & Q_{\zeta -1}≜\left(I-\left(TD_{\zeta -1}^{T}\right){\left(D_{\zeta -1}TD_{\zeta -1}^{T}\right)}^{-1}D_{\zeta -1}\right)T\\ \; & \left(I-D_{\zeta -1}^{T}{\left(D_{\zeta -1}TD_{\zeta -1}^{T}\right)}^{-1}\left(D_{\zeta -1}T\right)\right) \end{array}$$

□

## Appendix B. Proof of the (A3) and (A4)

We demonstrate this through the following process.

**Proof.** From (A1), we can find that the noise varianece:

(A7) $$\begin{array}{cc} \; & \sigma_{SE}^{2}\left(\left.\tau ,{\bar{a}}_{\zeta },\theta ,\rho ,P_{e}\right|D_{\zeta -1}\right)\\ \; & ≜{∥\tau ∥}^{2}+{∥\left(\tau^{T}{\left(G^{m}\right)}^{T}\theta +\rho^{T}D_{\zeta -1}-{\bar{a}}_{\zeta }^{T}\right)P_{e}^{\frac{1}{2}}∥}^{2}\\ \; & =\tau^{T}\left(I+{\left(G^{m}\right)}^{T}\theta P_{e}\theta G^{m}\right)\tau -2\tau^{T}{\left(G^{m}\right)}^{T}P_{e}\theta \left({\bar{a}}_{\zeta }-D_{\zeta -1}^{T}\rho \right)\\ \; & +\left(\rho^{T}D_{\zeta -1}-{\bar{a}}_{\zeta }^{T}\right)P_{e}\left({D_{\zeta -1}}^{T}\rho -{\bar{a}}_{\zeta }\right) \end{array}$$

Thus, the equalization vectors τ and ρ minimize the effective noise variance from (A7) can be used to find the extreme points by setting the derivative to 0. Firstly, this paper solve the vector τ. According to the (A7), we can solve for the first order derivative of an equation, and set it equal to zero, we obtain

(A8) $$\frac{\partial \sigma_{SE}^{2}}{\partial \tau }=\left(I+{\left(G^{m}\right)}^{T}\theta P_{e}\theta G^{m}\right)\tau -{\left(G^{m}\right)}^{T}P_{e}\theta \left({\bar{a}}_{\zeta }-D_{\zeta -1}^{T}\rho \right)$$

Let the $\frac{\partial \sigma_{SE}^{2}}{\partial \tau }=0$, the equation can be represented as

(A9) $$\tau^{opt}={\left(I+{\left(G^{m}\right)}^{T}\theta P_{e}\theta^{T}G^{m}\right)}^{-1}{\left(G^{m}\right)}^{T}P_{e}\theta \left({\bar{a}}_{\zeta }-D_{\zeta -1}^{T}\rho \right)$$

Then, plugging the $\tau^{opt}$ back to the (A8), we can obtain

(A10) $$\begin{array}{cc} \; & \sigma_{SE}^{2}\left(\left.\tau ,{\bar{a}}_{\zeta },\theta ,\rho \right|D_{\zeta -1}\right)\\ \; & =\left({\bar{a}}_{\zeta }^{T}-\rho^{T}D_{\zeta -1}\right)T\left({\bar{a}}_{\zeta }-D_{\zeta -1}^{T}\rho \right) \end{array}$$

where $T=P_{e}-\theta P_{e}G^{m}{\left(I+{\left(G^{m}\right)}^{T}\theta P_{e}\theta^{T}G^{m}\right)}^{-1}{\left(G^{m}\right)}^{T}P_{e}\theta$, Next, we solve the first order derivative for the equation (A10) and set its derivative function value to 0.

(A11) $$\frac{\partial \sigma_{SE}^{2}}{\partial \rho }={\bar{a}}_{\zeta }^{T}T{D_{\zeta -1}}^{T}-\rho^{T}D_{\zeta -1}TD_{\zeta -1}^{T}$$

Let $\frac{\partial \sigma_{SE}^{2}}{\partial \rho^{T}}=0$, it follows that

(A12) $${\rho^{opt}}^{T}=\left({\bar{a}}_{\zeta }^{T}TD_{\zeta -1}^{T}\right){\left(D_{\zeta -1}TD_{\zeta -1}^{T}\right)}^{-1}$$

□
